# Regulatory mechanisms and therapeutic implications of insulin-like growth factor 2 mRNA-binding proteins, the emerging crucial m^6^A regulators of tumors

**DOI:** 10.7150/thno.86528

**Published:** 2023-07-24

**Authors:** Heng Zhou, Qiang Sun, Mingliang Feng, Ziming Gao, Shiheng Jia, Lanxin Cao, Xue Yu, Shan Gao, Huizhe Wu, Kai Li

**Affiliations:** 1 Department of Surgical Oncology and General Surgery, Key Laboratory of Precision Diagnosis and Treatment of Gastrointestinal Tumors, Ministry of Education, The First Hospital of China Medical University, Shenyang, Liaoning 110001, People's Republic of China.; 2 Department of Anesthesiology, The First Hospital of China Medical University, Shenyang, Liaoning 110001, People's Republic of China.; 3 Department of Plastic Surgery, The First Hospital of China Medical University, Shenyang, Liaoning 110001, People's Republic of China.; 4 Department of Endoscopy, The First Hospital of China Medical University, Shenyang, Liaoning 110001, People's Republic of China.; 5 Department of Gynecology and Obstetrics, Shengjing Hospital of China Medical University, Shenyang, Liaoning 110001, People's Republic of China.; 6 Department of Pharmacology, School of Pharmacy, China Medical University, Shenyang, 110122, People's Republic of China.; 7 Liaoning Key Laboratory of Molecular Targeted Anti-Tumor Drug Development and Evaluation; Liaoning Cancer Immune Peptide Drug Engineering Technology Research Center; Key Laboratory of Precision Diagnosis and Treatment of Gastrointestinal Tumors, Ministry of Education; China Medical University, Shenyang, 110122, People's Republic of China.; 8 Shenyang Kangwei Medical Laboratory Analysis Co. LTD, Liaoning Province, China.

**Keywords:** Cancer, N^6^-methyladenosine, IGF2BPs, Reader, Cancer therapy

## Abstract

Insulin-like growth factor 2 mRNA-binding proteins (IGF2BPs) serve essential biological functions as post-transcriptional performers, participating in the acquisition or maintenance of tumor hallmarks due to their distinct protein structures. Emerging evidence indicates that IGF2BPs belong to the class III type of RNA N^6^-methyladenosine (m^6^A) modification readers, controlling RNA stability, storage, localization, metabolism, and translation in multiple vital bioprocesses, particularly tumorigenesis and tumor progression. Here, we discuss the underlying regulatory mechanisms and pathological functions of IGF2BPs which act as m^6^A readers in the context of tumor pathogenesis and multidrug resistance. Furthermore, we highlight the potential of IGF2BPs as drug targets in clinical tumor treatment. Hence, precise and novel tumor therapeutic approaches could be uncovered by targeting epigenetic heterogeneity.

## Introduction

Insulin-like growth factor 2 mRNA-binding proteins (IGF2BPs, IMPs), including IGF2BP1/2/3, are localized inside the nucleus as well as the cytoplasm. These proteins comprise two RNA recognition motifs (RRMs) and four K homology (KH) domains. In 1999, IGF2BP1/2/3 were first identified as a group of proteins that could potently bind to IGF2 leader 3 (IGF-II-L3) mRNA 1. To date, IGF2BPs have been found to be dysregulated in at least 15 types of tumors, such as gastric cancer (GC), colorectal cancer (CRC), and breast cancer, etc. Initially, IGF2BPs were identified by their ability to bind in vitro transcribed (unmodified) RNAs. In recent years, IGF2BPs have been found to also recognize the m^6^A modification on mRNAs and non-coding RNAs (ncRNAs), promoting their stability or translation and regulating the acquisition and maintenance of tumor hallmarks, such as cell proliferation 2, metabolic reprogramming 3, and immune evasion 4, etc. Diverse regulatory mechanisms, such as ncRNAs, transcription factors (TFs), and post-translational modifications (PTMs), manipulate the dysregulation of IGF2BPs in tumor initiation and development. Moreover, IGF2BPs have been implicated in the development of multi-drug resistance during tumor therapy 5 and can serve as prognostic predictors for the clinical outcomes of patients with tumors 6. Given the spectrum of oncogenes that these proteins can bind to, developing pharmaceutical inhibitors of IGF2BPs, including small-molecule inhibitors, represents a significant therapeutic avenue.

**Figure 1:**
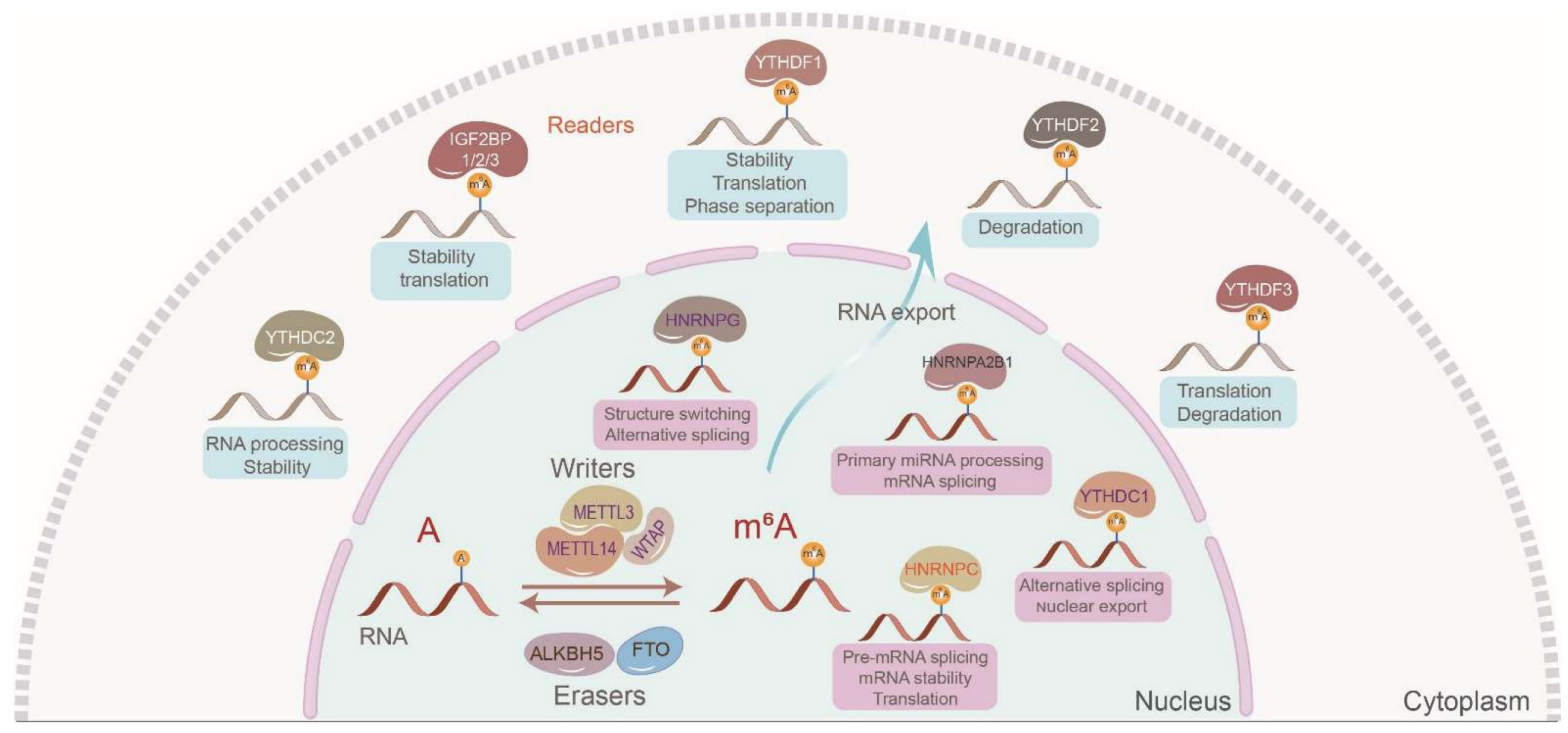
The canonical regulatory process of m^6^A modification. The dynamic and reversible m^6^A modification mostly occurs in the 3′ untranslated region (3′ UTR) and the vicinity of stop codons in target RNAs with a typical consensus sequence of “RRACH” (R = G or A; H = A, C, or U). m^6^A writers mainly include METTL3, METTL14, and WTAP which install the m^6^A marks. m^6^A erasers involving FTO and ALKBH5 demolish m^6^A marks. m^6^A readers mainly including IGF2BPs, hnRNPs, and YT521-B homology domain family recognize the m^6^A marks to manipulate localization, decay, stability, and translation of target RNAs.

In 2018, IGF2BPs were identified as a novel class III family of m^6^A readers governing the fate of downstream target RNAs in an m^6^A-dependent manner 7. Of the more than 170 types of modifications identified in mRNAs and ncRNAs, m^6^A is the most abundant in eukaryotes 8. The m^6^A modification mostly occurs in the 3′ untranslated region (3′ UTR) and the vicinity of stop codons in target RNAs with a typical consensus sequence of “RRACH” (R = G or A; H = A, C, or U). The canonical m^6^A regulatory process is dynamic and reversible. Generally, m^6^A writers including methyltransferase 3 (METTL3), methyltransferase 14 (METTL14), Wilms tumor 1-associated protein (WTAP), etc., m^6^A readers including IGF2BPs, heterogeneous nuclear ribonucleoproteins (hnRNPs), YT521-B homology (YTH) domain family, etc., and m^6^A erasers including fat mass and obesity-associated protein (FTO), alkB homolog 5 (ALKBH5), etc., create, recognize, and demolish m^6^A modifications on target RNAs, respectively 9, 10 (Figure 1). However, we should notice that very few adenosines are likely to be methylated in most RNAs, even in the 3'UTR of RNAs. Additionally, we should also notice that it is difficult to confirm the percent of the RNAs which are actually methylated among those RNAs that can be methylated.

Being novel m^6^A readers, IGF2BPs can control RNA stability, storage, localization, metabolism, and translation in multiple vital bioprocesses. Herein, we have outlined in detail the structures, functions, mechanisms, and therapeutic potential of IGF2BPs in oncology. This review provides insights into the m^6^A role of IGF2BPs in cancers and helps identify novel therapeutic strategies.

## Subcellular localization and structure of IGF2BPs

**Figure 2:**
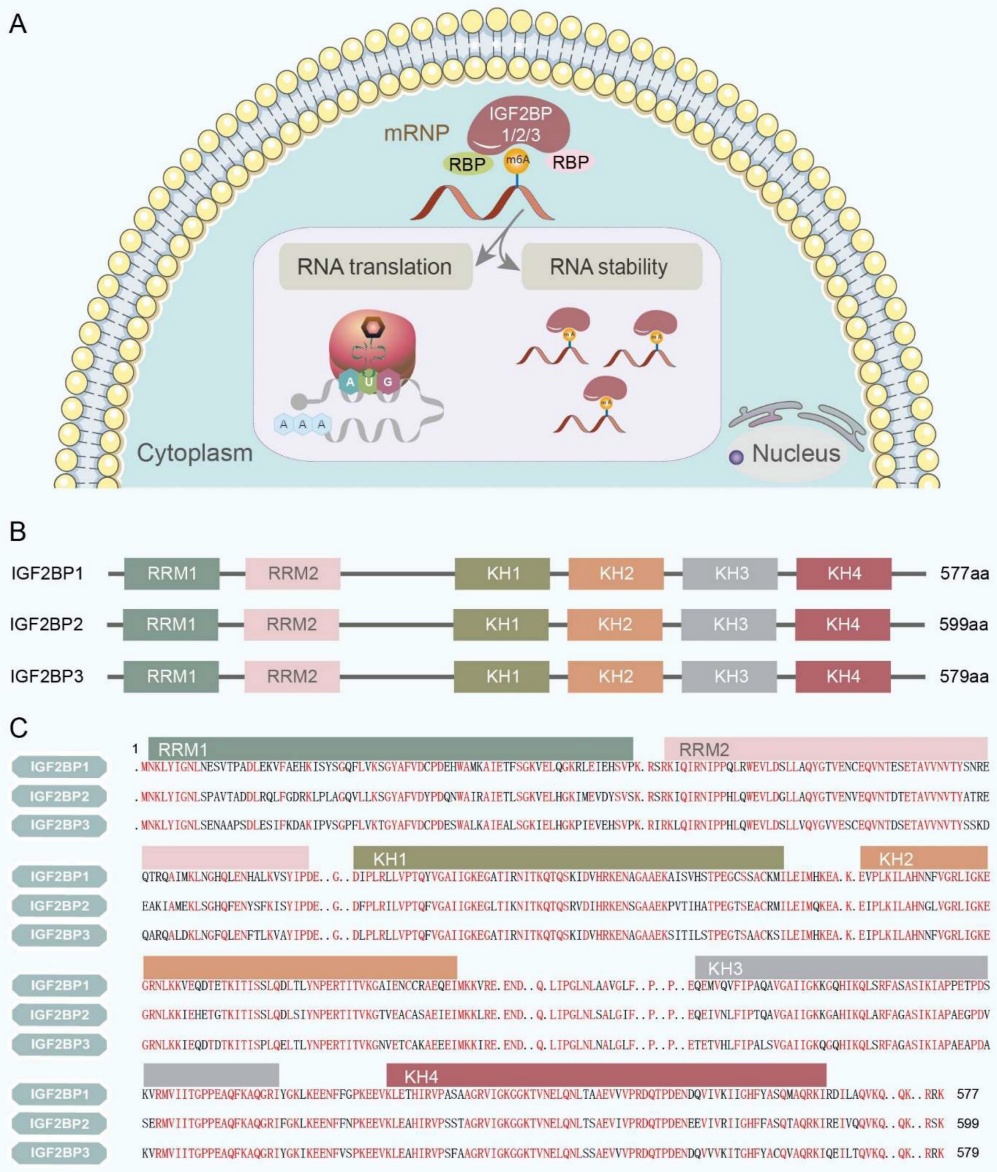
Subcellular localization and structure of IGF2BPs. **(A)** IGF2BPs are predominantly observed in the cytoplasm, where they 'cage' target mRNAs and ncRNAs within cytoplasmic RNPs. And they modulate a spectrum of RNA processes, including translation and stability, primarily through granule-like messenger ribonucleoprotein structures located in the perinuclear region. **(B)** IGF2BPs are highly conserved RNA-binding proteins that consist of two RRMs in the N-terminal region and four heterogeneous nuclear ribonucleoprotein KH domains in the C-terminal region. **(C)** The amino acid sequence of the IGF2BPs has been validated with over 56% similarity. Furthermore, IGF2BP1 and IGF2BP3 exhibit the closest similarity with 73% amino acid sequence identity. The sequence similarity of IGF2BPs is highlighted in red.

In humans, the subcellular localization of IGF2BPs ultimately determines their function and the fate of their target RNAs. IGF2BPs are predominantly observed in the cytoplasm, where they 'cage' target mRNAs and ncRNAs within cytoplasmic ribonucleoprotein complexes (RNPs). In the cytoplasm, IGF2BPs modulate a spectrum of RNA processes, including localization, translation, stability, and metabolism, primarily through granule-like messenger ribonucleoprotein structures located in the perinuclear region 9 (Figure 2A). In contrast, IGF2BPs have only marginally been detected in the nucleus 11.

Structurally, IGF2BPs are highly conserved RNA-binding proteins having two RRMs in the N-terminal region and four KH domains in the C-terminal region (Figure 2B). In mammals, their molecular weights range from 58-66 kDa 12. The amino acid sequences of the IGF2BPs have been validated with a similarity of over 56%. Furthermore, IGF2BP1 and IGF2BP3 exhibit the closest sequence identity (73%; Figure 2C). This extent of sequence similarity among IGF2BPs indicates that they perform analogical biochemical functions, including binding RNA, irrespective of the organism, tissue, or cell type 9.

*In vitro* studies have demonstrated that the KH domains primarily administrate RNA binding, whereas the RRM domains stabilize the IGF2BP-RNA complexes, leading to a prolonged half-life 13. Structural analysis has illustrated that the KH3 and KH4 domains of IGF2BP1 assume an anti-parallel pseudo-dimer conformation in which they contact the target RNA and are responsible for the transport and local translation of β-actin mRNA 14 and many other RNAs. Moreover, KH4 is found to identify a non-canonical “GGA” sequence by means of a broadened and dynamic hydrophobic groove, while KH3 binds to a central “CA” sequence with weak nucleotide discrimination 15. Relying on the representative KH3-4 domain, IGF2BPs can stabilize mRNAs; for example, overexpressed IGF2BP1 is shown to recognize the m^6^A sites in the 3' UTR of pyruvate kinase M1/2 (PKM2) via the KH3-4 domain to promote cancer progression^16^. Besides mRNAs, IGF2BPs can interact with ncRNAs through the KH3-4 domain; for instance, the KH3-4 domain of IGF2BP2 interacts with the “CAUCAU” m^6^A sequence motif at the exon 5-exon 4 junction of *circNSUN2* to promote colorectal liver metastasis 17. Moreover, IGF2BP2 activates the Warburg effect in CRC by recognizing the m^6^A modification on the long ncRNA (lncRNA) *ZFAS1* via the KH3-4 domain 18. The roles of the KH1-2 and RRM1-2 domains in RNA recognition are not clearly understood yet. A single recent study has revealed that IGF2BP1 fortifies the stability of its target RNA by binding to it via its KH1-2 domain. Specifically, IGF2BP1, through its KH1-2 domain, recognizes the METTL3-installed m^6^A on lncRNA *ABL* to maintain its stability and further inhibits apoptosome assembly and caspase‐9/3 activation in GC 19. These findings hint that the functions performed by the KH1-2 and KH3-4 domains may be identical.

Considering the unique biochemical functions of the IGF2BP domains, structure-based screening via docking to the KH domains may be a feasible strategy to identify potential IGF2BP inhibitors.

## The biological functions of IGF2BPs in tumors

**Figure 3:**
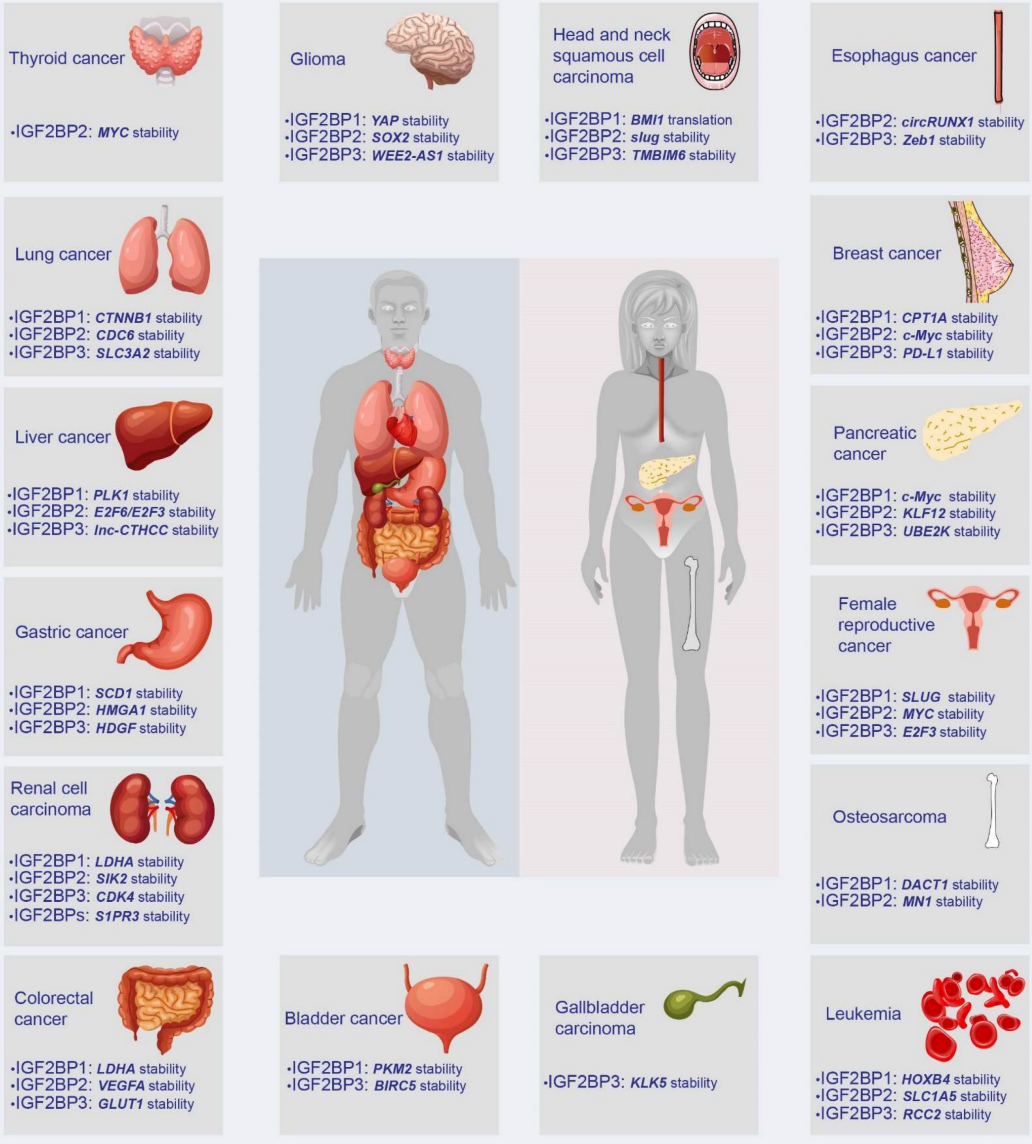
The expression and target genes of IGF2BPs in human tumors. In humans, IGF2BPs are overexpressed in at least 15 types of tumors, such as GC, CRC, liver cancer, and breast cancer. IGF2BPs promote the stability or translation of downstream target RNAs, including mRNA, lncRNA, and circRNA.

**Figure 4:**
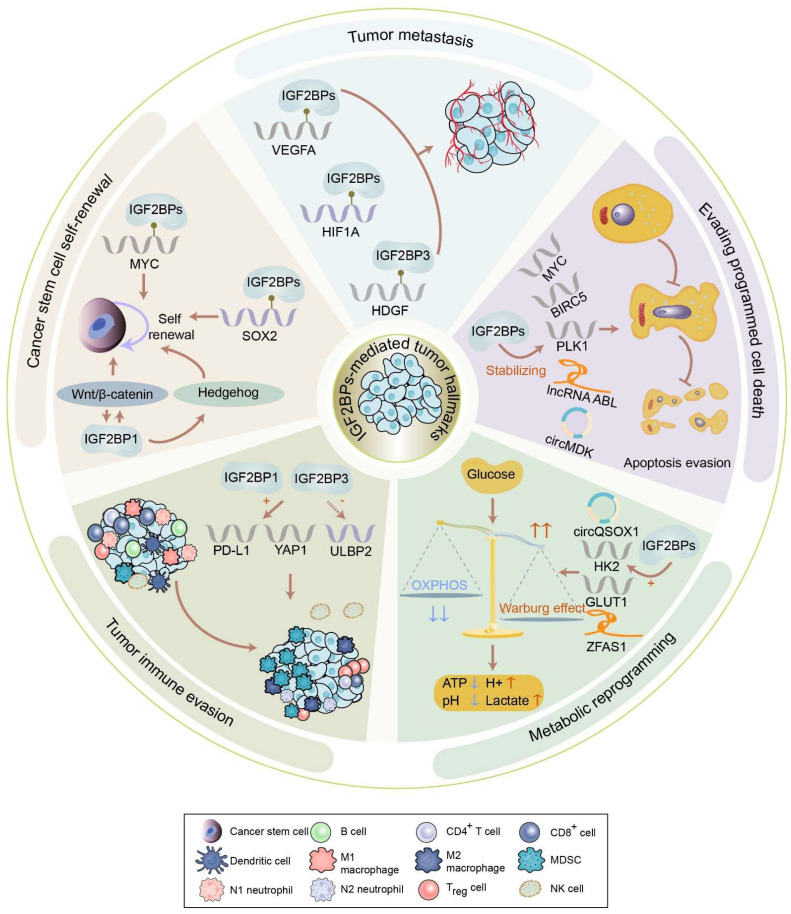
The biological functions of IGF2BPs in tumors. IGF2BPs participate in the acquisition or maintenance of tumor hallmarks, including cancer cell self-renewal, tumor angiogenesis, metabolic reprogramming, immune evasion, etc. IGF2BPs are involved in cancer cell self-renewal via managing cancer stemness-related genes and pathways. IGF2BPs induce tumor angiogenesis via stabilizing VEGFA, HIF1A, and HDGF. IGF2BPs stabilize apoptosis related RNAs, including mRNA, lncRNA, and circRNA, to facilitate tumor apoptosis evasion. IGF2BPs maintain the stability of HK2, GLUT1, circQSOX1, and ZFAS1 to activate the Warburg effect. IGF2BP1 and IGF2BP3 elevate the stability and expression of PD-L1, mediating immune evasion. IGF2BP3 diminishes the ability of natural killer cells to recognize transformed cells by downregulating ULBP2.

IGF2BPs are observed in most tissues during embryogenesis. Distinguishingly, IGF2BP2 is ubiquitously expressed in normal adult and tumor tissues, whereas IGF2BP1 and IGF2BP3 are *de novo* synthesized during numerous malignancies, earning themselves the label of bona fide oncofetal proteins. However, IGF2BP1 has also been found to play a tumor-suppressive role in multiple cancers. For instance, decreasing IGF2BP1 enhances the abilities of proliferation and migration of metastatic breast cancer cells 20. Moreover, loss of IGF2BP1 promotes cell proliferation of leukemia 21. Additionally, the knockdown of stromal IGF2BP1 facilitates a carcinogenic microenvironment and increases the histologic grade of colitis-associated cancer 22. This contradiction remains confusing. As oncogenes, IGF2BPs have been reported in various tumors, including breast cancer, GC, and CRC, among others (Figure 3). Accumulating evidence has linked IGF2BPs with tumorigenesis, tumor progression, the establishment of tumor cell hierarchy, and poor prognosis (Table 1). Notably, loss- and gain-of-function models have validated the effect of IGF2BPs on aggressive phenotypes of cancer, robustly evidencing their status as oncogenes influencing cancer cell self-renewal, angiogenesis, apoptosis, metabolic reprogramming, immune evasion, etc. (Figure 4).

### Cancer stem cell self-renewal

Self-renewal is a process of division to produce sufficient stem cells that can last throughout life. Remarkably, IGF2BPs play an indispensable role in facilitating the self-renewal of cancer stem cells. Of note, *MYC*, one of the most frequently activated oncogenes which contribute to the self-renewal of cancer stem cells, has been validated to be a significant target of IGF2BPs 7. *In vivo* and *in vitro* assays have illustrated that IGF2BPs can enhance the recognition of m^6^A on *MYC* in CRC, triple-negative breast cancer, GC, acute myeloid leukemia (AML), etc. 23-27. Specifically, IGF2BP1 maintains the stability of *MYC* by binding to the coding region determinant of m^6^A-modified *MYC* in a hypoxic microenvironment, thereby promoting the self-renewal of breast cancer stem cells 28. Moreover, the expression of SRY-box transcription factor 2 (*SOX2*), also essential for the self-renewal of cancer stem cells, is found to be governed by IGF2BPs. IGF2BP1 binds to the m^6^A sites in the 3′ UTR of *SOX2* to suppress its decay in endometrial cancer (EC) 29. Likewise, IGF2BP2 recognizes the coding sequence (CDS) of *SOX2* to prevent its degradation in CRC, thus reinforcing the self-renewal of stem cells 30. The IGF2BP3-mediated snail family transcriptional repressor 2 (*SLUG*) activates SOX2 transcription, contributing to the self-renewal of triple-negative breast cancer cells 31. By maintaining the stability of E2F transcription factor 6 (*E2F6*)/ E2F transcription factor 3 (*E2F3*), IGF2BP2 facilitates the stem cell self-renewal of liver cancer 32.

Besides managing cancer stemness-related genes, IGF2BPs also participate in cancer stemness-related signaling pathways, such as Wnt/β-catenin, Hedgehog, and Hippo signaling, to promote the self-renewal of cancer stem cells. Activation of the Wnt/β-catenin pathway is shown to sustain the self-renewal of stem cells 33, while Dishevelled-binding antagonist of β-catenin 1 (*DACT1*) is confirmed to negatively regulate this pathway 34. Interestingly, IGF2BP1 fails to stabilize *DACT1* after the m^6^A modification is eliminated by FTO, leading to the downregulation of *DACT1* and further stimulation of the Wnt/β-catenin pathway in osteosarcoma 35. Being a target of Wnt/β-catenin pathway, IGF2BP1 stabilizes GLI family zinc finger 1 (*GLI1*) to activate Hedgehog signaling in CRC and basal cell carcinoma cells 36, 37. Additionally, overexpression of IGF2BP3 inhibits Hippo signaling in a de-ubiquitination-dependent manner in gallbladder cancer 38.

Thus, IGF2BPs promote cancer stem cell self-renewal which accounts for cancer initiation, progression, recurrence, and metastasis, via related oncogenes and pathways. Targeting these proteins may provide a promising intervention to thwart the self-renewal capacity of cancer stem cells.

### Tumor metastasis

Metastasis is the most fatal manifestation of tumors. Of note, IGF2BPs can induce tumor metastasis via regulating tumor angiogenesis, epithelial-mesenchymal transition (EMT), etc. Tumor angiogenesis has been considered an effective therapeutic target for cancers because it critically supports tumor growth and metastasis by supplying urgent requirements of oxygen and nutrients. It is primarily driven by the vascular endothelial growth factor A (VEGFA)-related signal transduction pathway. Strikingly, IGF2BPs have been shown to promote tumor angiogenesis by stabilizing *VEGFA*. For instance, IGF2BP3 directly enhances the stability of m^6^A-modified *VEGFA* to promote angiogenesis in colon cancer 39. It also indirectly increases *VEGFA* expression by stabilizing apoptosis inhibitor Survivin (BIRC5), thus contributing to angiogenesis in bladder cancer 40. Similarly, IGF2BP2/3 manipulate the expression of ephrin type-A receptor 2 (*EphA2*) and *VEGFA* to facilitate vasculogenic mimicry in CRC 41. In addition to *VEGFA*, IGF2BP3 directly binds to and stabilizes hypoxia inducible factor 1 subunit alpha (*HIF1A*) to promote angiogenesis in GC 42. Likewise, IGF2BP2 indirectly regulates *HIF1A* and matrix metalloproteinase 14 (*MMP14*) to form vasculogenic mimicry in glioma 43. Additionally, IGF2BP3 consolidates the stability of hepatoma-derived growth factor (*HDGF*), and this secreted protein accelerates angiogenesis in GC 44.

IGF2BPs also induce EMT that stimulates metastasis. In intrahepatic cholangiocarcinoma, IGF2BP1 stimulates the AKT/matrix metalloproteinase 2 (MMP2) signaling pathway to facilitate tumor metastasis in an m^6^A-dependent manner 45. Furthermore, IGF2BP2 increases the stability of *SLUG* mRNA which is an EMT-related transcriptional factor via binding to its m^6^A site in the CDS, leading to lymphatic metastasis in head and neck squamous carcinoma (HNSCC) 46. Additionally, IGF2BP3-mediated m^6^A modification stabilizes minichromosome maintenance complex component (*MCM5*) mRNAs and activates the Notch signaling, leading to EMT and metastasis in lung adenocarcinoma 47.

Altogether, these studies demonstrate that IGF2BPs facilitate tumor angiogenesis and EMT, which are crucial for tumor metastasis. Inhibiting IGF2BP expression can simultaneously suppress tumor metastasis, providing a novel strategy for decelerating cancer progression.

### Evading programmed cell death

IGF2BPs can dysregulate the programmed cell death, chiefly involving apoptosis, ferroptosis, and autophagy, in cancer. Apoptosis is a programmed response for lysing and obliterating stressed, impaired, malignant, or infected cells in an organism 48. IGF2BPs have been found to be associated with apoptotic pathways, which dictate cell fate in cancers. For example, the aforementioned *MYC* and *BIRC5*, which are upregulated by IGF2BPs, can also inhibit cancer cell apoptosis. Moreover, IGF2BP1 elevates the expression of Polo like kinase 1 (*PLK1*), thus inhibiting the apoptosis of hepatocellular carcinoma (HCC) cells 49. Furthermore, IGF2BP1 maintains the stability of the lncRNA *ABL*, which binds to apoptotic protease-activating factor 1 (*APAF1*) and prohibits apoptosome assembly as well as caspase-9/3 activation, assisting in the evasion of apoptosis 19. Moreover, IGF2BP1 facilitates the translation of *circMAP3K4* into a novel peptide, circMAP3K4-455aa, which blocks the apoptosis of HCC cells by inhibiting the cleavage and nuclear distribution of the mitochondria-associated apoptosis-inducing factor 1 (AIF) 50. Additionally, IGF2BP1 stimulates the translation of cellular inhibitor of apoptosis 1 (cIAP1), which regulates caspase-8-mediated cell death in rhabdomyosarcomas 51. IGF2BP3 elevates the expression of regulator of chromosome condensation 2 (*RCC2*) in an m^6^A-dependent manner to suppress apoptosis in AML 52. Simultaneously, IGF2BP3 stabilizes and upregulates transmembrane BAX inhibitor-1-containing motif 6 (*TMBIM6*), which negatively regulates the intrinsic apoptotic signaling pathway in laryngeal squamous cell carcinoma 53. Interestingly, IGF2BP1 promotes PI3K/AKT/mTOR signaling pathway-mediated apoptosis evasion via a circMDK-miR-346/miR-874-3p-ATG16L1 regulatory axis in HCC 54. In summary, IGF2BPs empower cancer cells to obtain the ability to escape apoptosis.

Ferroptosis is a newly discovered form of iron-dependent cell death. IGF2BPs have been identified to be involved in ferroptosis. For example, IGF2BP1 increases the stability of solute carrier family 7 member 11 (*SLC7A11*) mRNA which is modified by METTL3 in an m^6^A-dependent manner to resist ferroptosis in hepatoblastoma 55. In hypopharyngeal squamous cell carcinoma, IGF2BP2-involved m^6^A modification stabilizes *NFE2L2/NRF2* mRNA, contributing to ferroptosis resistance 56. Likewise, IGF2BP3 as an m^6^A reader also participates in anti-ferroptosis. IGF2BP3 could stabilize a spectrum of anti-ferroptosis mRNA, including glutathione peroxidase 4 (*GPX4*), solute carrier family 3 member 2 (*SLC3A2*), acyl-CoA synthetase long chain family member 3 (*ACSL3*), and ferritin heavy chain 1 (*FTH1*), to inhibit ferroptosis in lung adenocarcinoma cells 57.

Autophagy is the process by which cells meet the cellular metabolism and renew the organelles to maintain homeostasis 58. However, the role of autophagy in cancer is dichotomous. Recently, accumulating reports have revealed that IGF2BPs participate in the regulation of autophagy during tumor progression. In clear cell renal cell carcinoma (ccRCC), IGF2BP2 stabilizes salt inducible kinase 2 (SIK2) mRNA to enhance autophagic flux via the FTO-mediated m^6^A modification, diminishing the ccRCC growth and metastasis 59. On the contrary, IGF2BP2 elevates the stability of a deubiquitinase ubiquitin specific peptidase 13 (*USP13*) to prolong the half-life of autophagy-related protein 5 (*ATG5*), inducing pro-survival autophagy and drug resistance in gastrointestinal stromal tumors 60. Dramatically, whatever the role autophagy plays in tumor development, IGF2BPs have consistently played the role of oncogenes.

Programmed cell death plays a vital role in inducing resistance to radiotherapy and chemotherapy. Therefore, inhibiting IGF2BPs may enhance the sensitivity toward radiotherapy and chemotherapy, improving the overall status quo of anti-tumor therapy resistance.

### Metabolic reprogramming

Metabolic reprogramming, mainly that of glucose, fatty acid, and amino acid metabolism, is a mechanism by which cells change their metabolic patterns to satisfy the energy needs, thereby promoting proliferation and growth. This process not only helps cells resist external stress but also endows them with new functions. It is frequently linked with tumorigenesis as well. Remarkably, IGF2BPs can influence metabolic reprogramming.

The Warburg effect or aerobic glycolysis is the most common pathway of metabolic reprogramming in tumors. *MYC*, which is amplified by IGF2BPs, helps enhance the Warburg effect in cancers 61-63. IGF2BPs promote glucose metabolism in cancer cells by stabilizing lactate dehydrogenase A (*LDHA*) 64, 65. Specifically, IGF2BP1 enhances glycolysis in GC cells by upregulating NADH dehydrogenase (ubiquinone) 1 alpha subcomplex 4 (*NDUFA4*) in an m^6^A-dependent manner 3. Likewise, IGF2BP1 also binds to the 3′ UTR of *PKM2* to induce the Warburg effect in bladder cancer 16. Moreover, IGF2BP2 recognizes and upregulates m^6^A-decorated Apolipoprotein E (*APOE*) to promote glycolysis and tumor growth in papillary thyroid cancer 66. Moreover, IGF2BP2-stabilized hexokinase 2 (*HK2*) and IGF2BP2/3-stabilized solute carrier family 2 member 1 (*SLC2A1, GLUT1*) activate glycolysis to promote CRC progression 67. Furthermore, IGF2BP3 directly recognizes *HDGF* in the nucleus to activate the expression of glucose transporter type 4 (*GLUT4*) and enolase 2 (*ENO2*), which enhances glycolysis in GC cells 44. Additionally, IGF2BP3 could mediate glycolysis in cancer cells by stabilizing pyruvate dehydrogenase kinase 4 (*PDK4*) 68.

Besides IGF2BPs-mediated mRNAs, ncRNAs can also regulate glucose metabolism in cancer cells. For instance, IGF2BP2 activates the Warburg effect by consolidating the *ZFAS1*/ Obg like ATPase 1 (*OLA1*) axis in CRC 18. circFOXK2 cooperates with IGF2BP3 to accelerate aerobic glycolysis in oral squamous cell carcinoma by stabilizing *GLUT1* 69. *circQSOX1*, which is stabilized by IGF2BP2, provokes glycolysis via the miR-326/miR-330-5p/ phosphoglycerate mutase 1 (*PGAM1*) axis in CRC 70.

Acetyl-CoA is an important intermediate metabolite in energy metabolism. Notably, it is a precursor for the biosynthesis of lipid molecules that support cancer cell growth and proliferation. IGF2BP3 sustains the cellular levels of acetyl-CoA by directly binding to lncRNA *TINCR* and preventing its degradation, which blocks the ubiquitin-mediated degradation of ATP citrate lyase to promote lipid biosynthesis and induces cancer progression and chemoresistance 71. Glutamine as an anaplerotic substrate replenishes the tricarboxylic acid cycle and enhances tumor proliferation. Remarkably, IGF2BP2 governs glutamine uptake and metabolism by upregulating *MYC*, glutamic-pyruvic transaminase 2 (*GPT2*), and solute carrier family 1 member 5 (*SLC1A5*) in AML, which indicates that IGF2BPs could regulate amino acid metabolism in cancers 26.

These studies highlight that IGF2BPs can influence the metabolic reprogramming of cancer cells in an m^6^A-dependent manner. Intervention of IGF2BPs may perturb the state of tumor cells and the tumor microenvironment, thereby inhibiting tumor growth.

### Tumor immune evasion

Immune evasion has emerged as a crucial phenomenon in cancer progression and immunotherapy resistance. Unsurprisingly, IGF2BPs modulate tumor immune surveillance as well. Of note, the aforementioned pathway of aerobic glycolysis can promote tumor immune evasion 72. IGF2BPs can also directly or indirectly regulate immune checkpoints to facilitate immune evasion. In HCC, IGF2BP1 not only reduces the infiltration of immune cells, such as CD4^+^ and CD8^+^ T cells, CD56^+^ natural killer cells, and F4/80^+^ macrophages but also increases the expression of programmed death-ligand 1 (*PD-L1*), which indicates that IGF2BP1 may act as a new anti-tumor therapeutic target by disrupting the tumor immune microenvironment 73. Moreover, IGF2BP1 elevates the stability and expression of *PD-L1*, mediating the immune evasion of bladder cancer 4. Similarly, IGF2BP3 impairs anti-tumor immunity via PD-L1-mediated T cell activation, exhaustion, and infiltration in breast cancer 74. Furthermore, IGF2BP3 diminishes the ability of natural killer cells to recognize transformed cells by downregulating the stress-induced ligands UL16 binding protein 2 (*ULBP2*) and major histocompatibility complex class I polypeptide-related sequence B (*MICB*), contributing to tumor immune evasion 75. Additionally, IGF2BP3 could accelerate the polarization of macrophage tumor-promoting phenotypes to mediate immune evasion 76.

Overall, tumor immunotherapy has achieved some success, but many patients remain insensitive to it. IGF2BPs enhance tumor immune evasion, suggesting that a combination of tumor immunotherapy and IGF2BP inhibitors might provide novel insights to overcome resistance to tumor immunotherapy.

## Dysregulation of IGF2BPs in tumors

**Figure 5:**
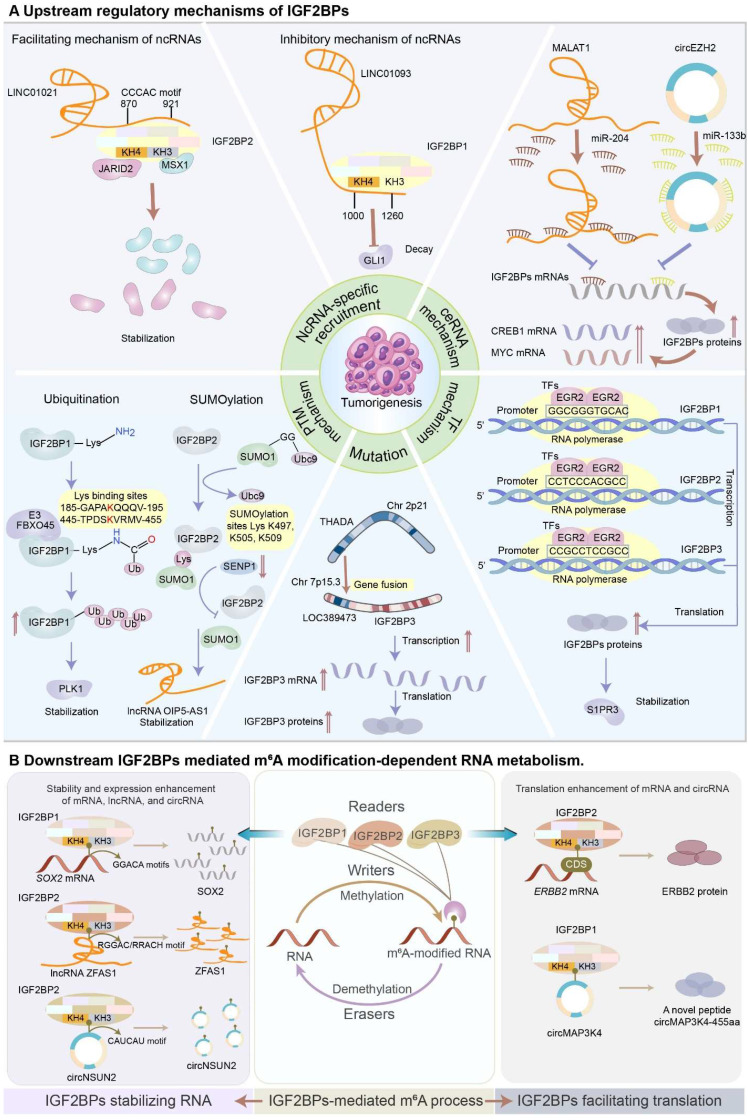
** (A)** Upstream regulatory mechanisms of IGF2BPs. **NcRNA-specific recruitment.** NcRNAs expedite or attenuate the function of IGF2BPs via ncRNA-specific recruitment. LINC01021 enhances the stability and expression of IGF2BP2 by recruiting it via the “CCCAC” fragment in the 870-921 nucleotide region, forming a LINC01021-IGF2BP2-MSX1/JARID2 signaling axis to promote CRC tumorigenesis and progression. Conversely, LINC01093 occupies the KH3-4 domain of IGF2BP1 via its 1000-1260-nucleotide region to decrease the binding affinity of IGF2BP1 to its target genes in HCC. **ceRNA mechanism.** IGF2BPs are manipulated by ceRNA machinery regulation at mRNA levels. LncRNA MALAT1 sponges miR-204 to upregulate IGF2BP2, contributing to the overexpression of MYC in an m^6^A-dependent manner. Similarly, circRNA circEZH2/ miR-133b/IGF2BP2/CREB1 axis orchestrates the CRC progression in an m^6^A-dependent manner. **TF mechanism.** TFs enhance the expression of IGF2BPs at transcriptional levels. EGR2 transcriptionally activates IGF2BPs by directly binding to the promoter regions to improve the expression of IGF2BPs, leading to the stabilization of S1PR3. **Mutation.** The gene fusion involving THADA and LOC389473 genes contributes to strong overexpression of IGF2BP3. **PTM mechanism.** IGF2BP proteins are governed by PTMs, predominantly including ubiquitination and SUMOylation, to perform multiple cellular processes. E3 ubiquitin ligase FBXO45 interacts with IGF2BP1 and facilitates its activation, leading to the stabilization of PLK1. SUMO1 SUMOylates IGF2BP2 at the lysine residues K497, K505, and K509, promoting cancer progression. **(B)** Downstream IGF2BPs-mediated m^6^A modification-dependent RNA metabolism. IGF2BPs recognize the m^6^A marks to increase the stability and expression of mRNAs, lncRNAs, and circRNAs. Mechanistically, IGF2BPs bind to the typical consensus motifs of RNAs by the unique KH3-4 domain to enhance the stability of RNAs. Additionally, IGF2BPs recognize the m^6^A marks to facilitate the translation of mRNAs and ncRNAs. IGF2BP2 binds to ERBB2 and promotes its translational efficiency. IGF2BP1 binds to circRNA circMAP3K4 and induces circMAP3K4 to translate into a novel peptide circMAP3K4-455aa.

In this section, we will mainly discuss the dysregulation of IGF2BPs during tumorigenesis and progression. These IGF2BPs are involved in multiple regulatory mechanisms, including ncRNA-specific recruitment, RNA-protein complex mechanism, competing endogenous RNA (ceRNA) regulation, transcriptional factor regulation, mutation regulation, protein post-translational modifications (Figure 5A), and manipulation of the fate of RNAs with m^6^A (Figure 5B).

### NcRNA-specific recruitment

#### NcRNAs expedite the function of IGF2BPs

Increasing evidence has indicated that ectopic lncRNAs and circRNAs exaggerate the function of IGF2BPs via an ncRNA-specific recruitment mechanism 77. Strikingly, by binding to IGF2BPs, lncRNAs prevent their degradation. Acting as a scaffold, an aberrantly overexpressed lncRNA, *KB-1980E6.3*, interacts with the KH1-2 domain of IGF2BP1 to constitute a *KB-1980E6.3*/IGF2BP1/c-Myc signaling axis, which promotes the stability of *c-Myc* and maintains the stemness of breast cancer stem cells 28. *LINC01021* enhances the stability and expression of IGF2BP2 by recruiting it via the “CCCAC” fragment in the 870-921 nucleotide region. Subsequently, upregulated IGF2BP2 binds to msh homeobox 1 (*MSX1*) and jumonji and AT-rich interaction domain containing 2 (*JARID2*) to form a *LINC01021*-IGF2BP2-*MSX1/JARID2* signaling axis, which contributes to CRC tumorigenesis and progression 78. The lncRNA *DMDRMR* recruits IGF2BP3 and enhances its stability to drive the progression of ccRCC. Mechanistically, the 51-115 nucleotide region in *DMDRMR* exon 1 binds to the KH1-2 domain of IGF2BP3 to form a *DMDRMR*/IGF2BP3/*CDK4* axis in ccRCC 2.

Likewise, circRNAs can also interact with IGF2BPs and enhance the function of RNA-binding proteins. For instance, circXPO1 is upregulated in lung adenocarcinoma and interacts with IGF2BP1 without influencing its expression to promote its function of stabilizing *CTNNB1*, leading to cancer progression 79. Upregulated circARHGAP29 interacts with IGF2BP2 to increase the expression of *LDHA*, which induces aerobic glycolysis in docetaxel-resistant prostate cancer via two different pathways 65. Firstly, *circARHGAP29* interacts with the KH3-4 domain of IGF2BP2 which binds to the “UGGAC” consensus sequence in the 3′ UTR of *LDHA*, directly forming a *circARHGAP29*-IGF2BP2-*LDHA* RNA-protein ternary complex in the cytoplasm to facilitate the stability of *LDHA* 65. Secondly, *circARHGAP29*, IGF2BP2, and *c-Myc* establish a novel RNA-protein ternary complex, which suppresses the decay of c-Myc protein and transcriptionally activates *LDHA* 65. Similarly, circARID1A acts as a scaffold to form a *circARID1A*-IGF2BP3-*SLC7A5* RNA-protein ternary complex in GC, which expedites the proliferation of GC cells 80.

#### NcRNAs attenuate the function of IGF2BPs

On the contrary, ncRNAs that exert antineoplastic effects can hinder the function of IGF2BPs. For instance, the lncRNA *FGF13-AS1* disrupts the association between IGF2BPs and their target mRNA by competitively binding to the “GGAC” m^6^A core motif to inhibit breast cancer progression 63. *LINC01093* interacting with the KH3-4 domain of IGF2BP1 via its 1000-1260 nucleotide region suppresses the binding affinity of IGF2BP1 to its target genes in HCC 81. The lncRNA *NBAT1* competitively binds to IGF2BP1 in HCC to impede its association with *MYC* 82.

Similarly, circRNAs can also perturb the oncogenic effect of IGF2BPs. *CircNDUFB2* acts as a scaffold to facilitate the binding between IGF2BPs and tripartite motif protein 25 (*TRIM25*), an E3 ligase, promoting the ubiquitination and degradation of IGF2BPs 83. Moreover, *circPTPRA* inhibits the progression of bladder cancer by occupying the KH3-4 domain of IGF2BP1, thus impairing its ability to recognize downstream m^6^A-modified mRNA 84. Furthermore, CA-rich sequences in the circRNA *CDR1as* interact with IGF2BP3 to attenuate the progression of melanoma 85. Additionally, acting as a protein decoy, *circTNPO3* directly binds to IGF2BP2/3 to suppress the metastasis of GC and ccRCC 24, 86.

Taken together, these findings suggest that the dualistic modulations between ncRNAs and IGF2BPs can influence tumorigenesis and cancer progression, offering innovative and precise cancer therapeutic approaches that target regulatory epigenetic characteristic biomarkers.

### ceRNA machinery regulation

miRNAs induce the degradation of target mRNAs or inhibit translation through direct reciprocal interactions 87. Remarkably, miRNAs or ceRNAs can regulate the expression of IGF2BPs to influence tumor progression. Numerous miRNAs have been identified as upstream regulators of *IGF2BP1*, including miR-372 88, miR-873 89, miR-494 90, and miR-885-5p 91. Moreover, miR-625, miR-196b, and miR-98-5p are shown to negatively regulate *IGF2BP1* by binding to its 3′ UTR, leading to the inhibition of malignant phenotypes in liver cancer 92-94. Similarly, miRNAs have been demonstrated to serve as tumor suppressors by modulating the expression of *IGF2BP2*. In HCC, miR-216b directly binds to the 3′ UTR of *IGF2BP2* to suppress cell growth 95. In lung cancer, overexpressed miR-485-5p inhibits growth and invasion by downregulating *IGF2BP2* 96. In CRC, *circEZH2*, acting as a miR-133b sponge, upregulates *IGF2BP2*, leading to cancer progression in an m^6^A-dependent manner 6. Likewise, the *MALAT1*/miR-204/IGF2BP2/m^6^A-*MYC* axis orchestrates the proliferation, migration, and invasion of thyroid cancer 97. As expected, miRNAs have also been shown to attenuate the pro-tumor biological functions of *IGF2BP3*. In GC, miR-34a suppresses cell proliferation and invasion by downregulating *IGF2BP3* 98. Notably, ceRNA signaling involving *circIGHG*/miR-142-5p/IGF2BP3 plays a critical oncogenic role in oral squamous cell carcinoma 99. Although developing miRNA-targeting anti-tumor drugs is tough currently, they are promising therapeutic targets.

Overall, these studies reveal that miRNAs also govern the mRNA expression of *IGF2BPs*. Therefore, manipulating the miRNAs which act as the upstream regulatory molecules of *IGF2BP*s might inhibit tumor malignancy.

### IGF2BPs regulated by Transcriptional factors

TFs control chromatin and transcription by recognizing specific DNA sequences to guide gene expression. TFs can enhance the expression of *IGF2BP*s. In renal cell cancer, the transcription factor early growth response protein 2 (EGR2) remarkably enhances *IGF2BP* levels by directly binding to the promoter regions, thereby driving tumorigenesis and metastasis 100. The transcription of *IGF2BP*s can be stimulated by different TFs separately. For instance, transcription factor-activating and enhancer-binding protein 4 (TFAP4) binds to the *IGF2BP1* promoter to activate its transcription in non-small cell lung cancer 101. Interestingly, IGF2BP1 stabilizes Yes1 associated transcriptional regulator (*YAP*) mRNA, leading to the activation of YAP/WW domain containing transcription regulator 1 (TAZ, WWTR1) signaling in an m^6^A-dependent manner. Reciprocally, TAZ induces the expression of IGF2BP1, creating a feedback loop to facilitate tumorigenesis and the progression of malignancy 102. Furthermore, Hepatocyte nuclear factor 4 gamma (HNF4G), acting as a TF, enhances the expression of *IGF2BP2* 103. Likewise, MYC effectively activates the expression of *IGF2BP3* by binding to its promoter to drive tumor progression and metastasis 104. Intriguingly, IGF2BP3 could stabilize *MYC* and increase its expression, establishing a positive feedback mechanism to expedite tumor deterioration.

Collectively, these findings illustrate that TFs influence the expression of *IGF2BP*s by binding to their promoter regions, adding to the complexity of the regulatory mechanisms governing IGF2BP expression.

### IGF2BPs regulated by Protein post-translational modifications

Protein post-translational modifications (PTMs) increase the functional diversity of the proteome and participate in multiple cellular processes. Undoubtedly, IGF2BPs, being RNA-binding proteins, are regulated by PTMs, predominantly involving ubiquitination and SUMOylation. Ubiquitination selectively degrades target proteins through the ubiquitin-proteasome system (UPS). However, an atypical E3 ubiquitin ligase, F-box protein 45 (FBXO45), can prohibit the proteolysis of some proteins. For instance, overexpressed FBXO45 interacts with IGF2BP1 and facilitates its activation, leading to liver tumorigenesis 49. Contrarily, tripartite motif-containing protein 21 (TRIM21), another E3 ubiquitin ligase, expedites IGF2BP3 decay via the UPS, arresting the growth of CRC 105. SUMOylation can facilitate numerous pivotal physiological and pathological processes by helping maintain protein stability. SUMOylation improves the expression of IGF2BP2 and prevents its UPS-mediated degradation. Mechanistically, IGF2BP2 is identified to be SUMOylated at the lysine residues K497, K505, and K509 by small ubiquitin-related modifier 1 (SUMO1), promoting the progression of cancer 43.

The use of proteolysis targeting chimera (PROTAC) is a revolutionary, emerging strategy for targeted protein degradation. Technically, PROTAC degrades target proteins through the UPS. PROTAC may tag IGF2BPs for degradation to attenuate cancer progression, offering a new approach for anti-tumor treatment.

### Mutation regulation on IGF2BPs

Mutations have been identified as crucial drivers in tumors. As expected, IGF2BPs can be governed by mutations mainly including gene fusion and copy number variation (CNV). In thyroid cancer, a recurrent fusion between the thyroid adenoma-associated (*THADA*) gene on chromosome 2 and the LOC389473 gene on chromosome 7 located 12 kb upstream of the IGF2BP3 gene contributes to high expression of IGF2BP3, activating the insulin like growth factor 1 receptor (*IGF1R*) signaling and facilitating the proliferation, invasion, and transformation of thyroid cancer cells 106. In pancreatic cancer, genomic mutation analysis based on the cBioPortal database reveals that the IGF2BP2 locus is amplified in 15.25% of patients, indicating that the overexpression of IGF2BP2 is highly correlated with CNV 107. These evidence shows that overexpression of IGF2BPs manipulated by gene fusion and amplification may become novel anti-tumor targets.

### RNA m^6^A modification-dependent metabolism

#### IGF2BPs enhancing RNA stability

The binding of IGF2BPs to mRNAs can help facilitate mRNA stability, which is predominantly mediated by aberrant writers or erasers in cancer 59, 60, 108. For example, in bladder cancer, IGF2BP1 recognizes the m^6^A moiety in the vicinity of the *PD-L1* stop codon, enhancing mRNA stability to induce immune escape via a METTL3-mediated m^6^A mechanism 4. In CRC, IGF2BP2 mainly binds to the CDS of *SOX2* to facilitate tumor progression in a METTL3-dependent manner 30. Similarly, IGF2BP3 recognizes the m^6^A signal added by METTL3 in the 3′ UTR of *BIRC5* mRNA to stabilize it, resulting in cell growth and metastasis of bladder cancer 40. Additionally, IGF2BPs also participate in the m^6^A process mediated by abnormally expressed m^6^A erasers. For instance, the depletion of FTO increases the binding propensity of IGF2BP2 toward metastasis-associated protein 1 (*MTA1*) mRNA, leading to CRC metastasis 109. Notably, IGF2BPs acting as m^6^A readers have been shown to maintain or enhance the stability of their target transcripts through their KH3-4 domain. Specifically, IGF2BP2 is confirmed to bind *MSX1* and *JARID2* through its KH3-4 domain in CRC 78. Moreover, the stabilizing activity of KH3-4 is limited. Once the KH3-4 domain is binding to an RNA, the ability to bind to other RNAs is significantly reduced. Research has shown that *circFAM13B* competes with *PKM2* to bind to the KH3-4 domain of IGF2BP1, leading to the attenuation of *PKM2* expression in bladder cancer 16.

NcRNAs such as lncRNAs, circular RNAs (circRNAs), and microRNAs (miRNAs) are critical regulators of tumor initiation and progression 110. IGF2BPs rely on their characteristic KH domains to recognize the m^6^A signals on ncRNAs, thereby sustaining their stability 111. IGF2BP1, with the help of its KH1-2 domain, recognizes and binds to the “GGACCACA” motif in lncRNA *ABL* to maintain its stability, consequently inhibiting the apoptosis of GC cells 19. IGF2BP2, depending on its KH3-4 domain, recognizes the m^6^A modification at adenosine +843 within the “RGGAC/RRACH” element of lncRNA *ZFAS1* to activate the Warburg effect in CRC 18. Furthermore, IGF2BP1 and IGF2BP3 recognize the “GGAC” site within *lnc-CTHCC* and prevent its decay in an m^6^A-dependent manner, thereby promoting hepatocellular carcinogenesis 112. Besides lncRNAs, overexpressed IGF2BP1 could bind to *circMDK* via its predicted “RRACU” m^6^A motif at the exon 5 site to augment the stability of this circRNA, subsequently promoting the progression of HCC 54. The KH3-4 domain of IGF2BP2 specifically interacts with the “CAUCAU” motif at the exon 5-exon 4 junction of *circNSUN2* to form an RNA-protein ternary complex, inducing CRC metastasis 17. Furthermore, IGF2BP3 increases the stability of circCCAR1 via a WTAP-dependent m^6^A modification to aggravate HCC progression 113.

Taken together, IGF2BPs function as the real executors in the m^6^A process. KH domains, especially the KH3-4 domain, play a vital role in identifying the m^6^A site. Disturbing the expression or activity of IGF2BPs may counter aberrant m^6^A process-induced tumorigenesis.

#### IGF2BPs facilitating RNA translation

In addition to increasing RNA stability, IGF2BPs also promote the translation of RNAs, including ncRNAs 50, 108. In oral squamous cell carcinoma, IGF2BP1 cooperates with METTL3 to promote *BMI1* translation by recognizing the “AUGGAC” motif in the 3′ UTR of *BMI1* 108. In papillary thyroid cancer, IGF2BP2 enhances the translation efficacy of erb-b2 receptor tyrosine kinase 2 (*ERBB2*) by binding to m^6^A motifs in its CDS, acquiring resistance to tyrosine kinase inhibitors 114. In osteosarcoma, IGF2BP2 interacts with m^6^A motifs in the CDS of *MN1* to promote both mRNA stability and translation 115. Notably, IGF2BP1 facilitates the translation of *circMAP3K4* into a novel peptide of 63 kDa, circMAP3K4-455aa, which inhibits the apoptosis of HCC cells. Mechanistically, m^6^A mutations at A862C and A787/862C of circMAP3K4 dramatically attenuate the binding affinity of IGF2BP1 and decrease the expression of circMAP3K4-455aa, indicating that IGF2BP1 modulates *circMAP3K4* translation via a METTL3-dependent m^6^A modification 50.

In summary, these findings highlight the role of IGF2BPs in RNA translation and reveal a novel carcinogenic pattern mediated by IGF2BPs, indicating that these proteins could be potential therapeutic targets in tumors.

## Targeting IGF2BPs as potential therapeutic biomarkers in tumors

IGF2BPs not only promote cancer progression but also blunt the sensitivity to anti-tumor therapies. In a nutshell, IGF2BPs expedite the development of drug resistance by enhancing tumor stemness 5, 116, blocking apoptosis 19, inducing metabolic reprogramming 16, 64, 70, etc. Logically, it follows that targeting IGF2BPs may be a novel anti-tumor strategy. More researches are required, though, to answer whether IGF2BPs are targetable. Remarkably, a few IGF2BP inhibitors have been screened out from the compound library (Table 2).

So far, merely four IGF2BP1 inhibitors have been discovered. The most representative of them is the small-molecule inhibitor 2-{[(5-bromo-2-thienyl) methylene]amino} benzamide (BTYNB), identified in 2017. It selectively decreases the expression of the mRNA targets of IGF2BP1, including *MYC*, by limiting the association between IGF2BP1 and the coding region stability determinants of its target mRNAs, eventually suppressing the proliferation of melanomas and ovarian cancer cells 117. Recently, BTYNB has shown the greatest efficacy in attenuating tumorigenesis and the growth of solid tumors by disrupting the interaction between IGF2BP1 and E2F-driven genes without influencing the abundance of the former 118. Furthermore, BTYNB is shown to suppress the growth of intrahepatic cholangiocarcinoma by decreasing the expression of *MYC* in a patient-derived xenograft model 45. C646, another small-molecule inhibitor, is identified to mitigate the mRNA and protein expression of IGF2BP1 in intrahepatic cholangiocarcinoma by inhibiting the levels of H3K27ac in its promoter 45. Recently, a high-throughput screen of over 27,000 small molecules leads to the discovery of a new IGF2BP1 inhibitor called '7773'. '7773' is confirmed to directly bind to the KH3-4 domain of IGF2BP1, weakening its binding affinity to Kras and attenuating the oncogenic effect of IGF2BP1 in cancer cells 119. Additionally, the fourth small-molecule inhibitor, cucurbitacin B, is considered to specifically recognize the Cys253 site in the KH1-2 domain of IGF2BP1, which allosterically impairs the ability of IGF2BP1 to read m^6^A signals in RNAs, promoting apoptosis and activating immune responses in HCC 73.

Similarly, IGF2BP2 inhibitors are woefully scarce, the first one being discovered as recently as in 2022. Ten compounds belonging to the benzamidobenzoic acid class and ureidothiophene class are screened out using a fluorescence polarization assay. Furthermore, partial compounds are confirmed to interact with the RRM1 and KH3-4 domains of IGF2BP2 and competitively inhibit its RNA-binding abilities without disrupting its expression in CRC. Meanwhile, the three most active compounds are validated to inhibit tumor growth *in vivo*. All these findings indicate that IGF2BP2 is a druggable anti-tumor target 120. Thereafter, a molecular docking model is applied to screen small-molecule compounds that could bind to the KH3-4 domain based on the three-dimensional structure of IGF2BP2. Resultantly, JX5, a novel small-molecule inhibitor of IGF2BP2, is identified. JX5 directly binds to IGF2BP2 without influencing its mRNA levels; instead, the inhibitor dramatically attenuates the expression of its downstream target RNA and decelerates the progression of T cell acute lymphoblastic leukemia 121. A new IGF2BP2 inhibitor named CWI1-2, purified from the compound NSC69557, has been demonstrated to reduce the binding affinity of IGF2BP2 to its RNA targets. Molecular docking illustrates that CWI1-2 docks to the hydrophobic pocket within the KH4 domain and competes with RNAs to bind to IGF2BP2. It could potentially exhibit anti-tumor properties in AML 122.

Unlike IGF2BP1 and IGF2BP2 inhibitors, the mechanism of action of IGF2BP3 inhibitors has not been clarified yet. Nevertheless, a few inhibitors influencing IGF2BP3 expression have been discovered. JQ1 123 and I-BET151 27, belonging to the bromodomain and extraterminal domain class of inhibitors, have been validated to downregulate IGF2BP3 expression and impair tumor growth in Ewing sarcoma and mixed-lineage leukemia-rearranged B-acute lymphoblastic leukemia. Berberine, an isoquinoline alkaloid derived from Coptidis Rhizoma, inhibits the proliferation of CRC cells by targeting IGF2BP3 124. Similarly, isoliquiritigenin, a flavonoid primarily obtained from licorice root, restrains the malignant phenotype of lung cancer cells by downregulating IGF2BP3 125.

Rigosertib (RIG), another small-molecule IGF2BP3 inhibitor, is identified by screening a library consisting of more than 1800 small molecules approved by the Food and Drug Administration. RIG represses IGF2BP3 expression and sensitizes lung cancer ​cells to RSL3 and erastin-induced ferroptosis, suggesting that RIG can inhibit tumor growth and promote programmed cell death 57.

In general, the development of virtual high-throughput screening and molecular docking has enabled the discovery of small-molecule inhibitors of IGF2BPs, which play antitumor roles mainly by inhibiting protein expression and activity. However, this class of inhibitors faces many limitations and challenges, such as the development of resistance to small-molecule inhibitors, the unsustainable inhibition of target protein activity, and “untargetable” proteins. The application of new technologies like PROTAC and molecular glue may overcome the limitations faced by small-molecule inhibitors. Thus, promising antitumor drugs targeting IGF2BPs may be screened out via PROTAC and molecular glue technology, providing a new strategy to treat tumors.

## Conclusions and perspective

The novel m^6^A readers IGF2BPs are widely overexpressed in cancers and participate in the regulation of mRNA stabilization and translation. IGF2BPs directly bind to m^6^A-modified RNAs via their KH domains, which are evolutionarily conserved RNA recognition elements. Dramatically, each IGF2BP can recognize more than 3,000 mRNA transcripts and at least 5,000 mRNAs, with great overlaps among the three IGF2BPs, suggesting that these proteins prominently govern m^6^A-dependent gene regulation 7. In addition to mRNAs, IGF2BPs also recognize ncRNAs, including lncRNAs and circRNAs, to exacerbate cancer progression. Intriguingly, the m^6^A modification, as a type of non-mutational epigenetic reprogramming, participates in the administration of most other cancer hallmarks partially via IGF2BPs. Emerging evidence has consistently demonstrated that IGF2BPs help cancer cells proliferate, invade tissues, metastasize, sustain angiogenesis, evade programmed cell death, dysregulate energy metabolism, and avoid immune destruction. All the evidence indicates that IGF2BPs may be promising druggable targets in tumor therapies. Several IGF2BP inhibitors have been proposed so far. Some directly interact with the KH domains to interfere with the RNA binding capacity without affecting IGF2BP expression, while the mechanism of others is still unclear and needs further research. The expression of IGF2BPs is also regulated by multiple mechanisms, mainly including ncRNAs, TFs, and PTMs, which can further manipulate the fate of oncogenes during tumorigenesis and tumor growth. The expression of IGF2BPs may be suppressed by interfering with their upstream regulatory mechanisms, eventually inhibiting tumor progression, which presents a novel antitumor approach. The discovery of IGF2BP inhibitors and the determination of upstream regulatory mechanisms both demonstrate the potential accessibility of IGF2BPs via exogenous manipulations.

In conclusion, IGF2BPs are crucial factors in tumor progression, chemotherapy resistance, and immunotherapy response. Therefore, incorporating a combination of IGF2BP inhibitors in antitumor therapy may remarkably inhibit cancer invasion, metastasis, and recurrence, or effectively overcome antitumor drug resistance. Unfortunately, fundamental research on IGF2BP inhibitors and their mechanisms is currently lacking, and no such clinical trial has ever been performed. Therefore, multidisciplinary collaboration is necessary to develop and optimize the use of IGF2BP inhibitors in cancer treatment.

## Figures and Tables

**Table 1:** The expression, function, and prognosis of IGF2BPs in cancers

IGF2BPs	Cancer type	Expression	Prognosis	Target genes or pathways	Biological function		Ref
**IGF2BP1**	Bladder cancer	Upregulated	Unfavorable	*MYC* and* FSCN1*	Promote cell proliferation, migration, and invasion.		84
	Intrahepatic cholangiocarcinoma	Upregulated	Unfavorable	*c-Myc* and* ZIC2*	Promote tumor growth and metastasis, inhibit senescence.		45
	Breast cancer	Upregulated	Unfavorable	*CPT1A*	Promote tumor metastasis.		126
	Gastric cancer	Upregulated	Unfavorable	*c-Myc*	Accelerate tumor aerobic glycolysis.		127
	Colorectal cancer	Upregulated	Unfavorable	*LDHA*	Promote glucose metabolism.		64
	Lung cancer	Upregulated	Unknown	*LIN28B*	Regulate cell cycle, DNA damage repair, and genome instability. Promote tumor proliferation and metastasis.		128
	Liver cancer	Upregulated	Unfavorable	*PLK1*	Promote tumor cell proliferation.		49
	Glioblastoma	Upregulated	Unfavorable	*YAP*	Promote stemness, sphere formation, and tumorigenicity.		102
	Endometrial cancer	Upregulated	Unfavorable	*PEG10*	Accelerate tumor cell proliferation and cell cycle progression.		129
**IGF2BP2**	AML	Upregulated	Unfavorable	*MYC, GPT2,* and* SLC1A5*	Promote tumor initiation/progression, stem cell self-renewal, and amino acid metabolism.		122
	ESCC	Upregulated	Unknown	*circRUNX1*	Promote tumor growth and metastasis.		130
	HNSCC	Upregulated	Unfavorable	*Slug*	Promote lymphatic metastasis and epithelial-mesenchymal transition.		46
	Colorectal cancer	Upregulated	Unfavorable	*ZFAS1*	Activate the Warburg effect.		18
	Glioma	Upregulated	Unknown	*OIP5-AS1*	Promote vasculogenic mimicry.		43
	Cervical cancer	Upregulated	Unknown	*MYC*	Accelerate tumor aerobic glycolysis.		62
	Pancreatic cancer	Upregulated	Unfavorable	*PI3K-Akt* signaling	Promote tumor cell proliferation.		107
	Gastric cancer	Upregulated	Unfavorable	*HMGA1*	Promote epithelial to mesenchymal transition and metastasis.		131
	Liver cancer	Upregulated	Unknown	*FEN1*	Promote tumor growth.		132
**IGF2BP3**	Lung cancer	Upregulated	Unfavorable	*GPX4, SLC3A2, ACSL3,* and* FTH1*	Suppress ferroptosis.		57
	Gastric cancer	Upregulated	Unfavorable	*SLC7A5*	Promote tumor proliferation.		80
	Nasopharyngeal carcinoma	Upregulated	Unfavorable	*KPNA2*	promote proliferation and metastasis.		104
	AML	Upregulated	Unfavorable	*RCC2*	promote proliferation and tumorigenesis.		52
	EC	Upregulated	Unfavorable	*E2F3*	Promote tumor growth.		133
	Colon cancer	Upregulated	Unfavorable	*CCND1* and* VEGF*	Accelerate cell cycle and angiogenesis.		39
**IGF2BPs**	Renal cell cancer	Upregulated	Unfavorable	*S1PR3*	Drive tumorigenesis and metastasis.		100

**Table 2:** Small molecule inhibitor of IGF2BPs

IGF2BPs	Inhibitors	Cancer types	Target site	Effect	Ref
**IGF2BP1**	BTYNB	Melanoma, Ovarian cancer	Unknown	Disrupting the interaction between	117, 118
				IGF2BP1 and target mRNAs	
	7773	Lung cancer	A hydrophobic surface at the	Reducing the level of mRNA targets	119
			boundary of KH3 and KH4 domain		
	Cucurbitacin B	HCC	The Cys253 site in the KH1-2 domain	A pharmacological allosteric effect to inhibit	73
				recognition of target mRNAs	
	C646	Intrahepatic	H3K27ac in the promoter of IGF2BP1	Decreasing the IGF2BP1 mRNA and protein	45
		cholangiocarcinoma		expression	
**IGF2BP2**	Ten compounds,	CRC, Liver cancer	RRM1 and KH3-4 (Compounds	Reducing tumor cell proliferation	120
	belonging to the		from benzamidobenzoic acid class),		
	benzamidobenzoic acid class		Unknown (Compounds from		
	and ureidothiophene class		ureidothiophene class)		
	JX5	Leukemia	KH3-4 domain	Suppressing the cancer progression without	121
				influencing the IGF2BP2 mRNA levels	
	CWI1-2	Leukemia	The hydrophobic pocket within	Decreasing downstream RNA and protein levels of	122
			the KH4 domain	IGF2BP2 to exert anti-tumor efficacy	
**IGF2BP3**	JQ1 and I-BET151	Leukemia,	Unknown	Decreasing levels of IGF2BP3 and its mRNA	27, 123
		Ewing Sarcoma		targets to hinder tumor growth	
	Berberine	CRC	Unknown	Down-regulating IGF2BP3 at the protein level to	105, 124
				inhibit CRC growth	
	Isoliquiritigenin	Non-small cell lung cancer	Unknown	Reducing IGF2BP3 at the mRNA and protein level	125
				to inhibit lung cancer progression	
	RIG	Lung adenocarcinoma	Unknown	Reducing IGF2BP3 expression and tumor growth	57

## Abbreviations

AIF: apoptosis-inducing factor 1
ALKBH5: alkB homolog 5
ACSL3: acyl-CoA synthetase long chain family member 3
AML: acute myeloid leukemia
APAF1: apoptotic protease-activating factor 1
APOE: Apolipoprotein E
ATG5: autophagy-related protein 5
BIRC5: apoptosis inhibitor Survivin
BTYNB: 2-{[(5-bromo-2-thienyl) methylene]amino} benzamide
ccRCC: clear cell renal cell carcinoma
CDS: coding sequence
ceRNA: competing endogenous RNA
cIAP1: cellular inhibitor of apoptosis 1
CNV: copy number variation
CRC: colorectal cancer
DACT1: Dishevelled-binding antagonist of β-catenin 1
EC: endometrial cancer
EMT: epithelial-mesenchymal transition
ENO2: enolase 2
EphA2: ephrin type-A receptor 2
FTH1: ferritin heavy chain 1
FTO: fat mass and obesity-associated protein
GC: gastric cancer
GLI1: GLI family zinc finger 1
GLUT4: glucose transporter type 4
GPT2: glutamic-pyruvic transaminase 2
GPX4: glutathione peroxidase 4
HDGF: hepatoma-derived growth factor
HIF1A: hypoxia inducible factor 1 subunit alpha
hnRNPs: heterogeneous nuclear ribonucleoproteins
HNSCC: head and neck squamous carcinoma
IGF2BPs: Insulin-like growth factor 2 mRNA-binding proteins
IGF-II-L3: IGF2 leader 3
JARID2: jumonji and AT-rich interaction domain containing 2
KH: K homology
LDHA: lactate dehydrogenase A
m^6^A: N6-methyladenosine
MCM5: minichromosome maintenance complex component
METTL14: methyltransferase 14
METTL3: methyltransferase 3
MICB: major histocompatibility complex class I polypeptide-related sequence B
MMP14: matrix metalloproteinase 14
MMP2: matrix metalloproteinase 2
MSX1: msh homeobox 1
ncRNAs: non-coding RNAs
NDUFA4: NADH dehydrogenase (ubiquinone) 1 alpha subcomplex 4
OLA1: Obg like ATPase 1
PDK4: pyruvate dehydrogenase kinase 4
PD-L1: programmed death-ligand 1
PGAM1: phosphoglycerate mutase 1
PKM2: pyruvate kinase M1/2
PLK1: Polo like kinase 1
PROTAC: proteolysis targeting chimera
PTMs: post-translational modifications
PTMs: post-translational modifications
RCC2: regulator of chromosome condensation 2
RNPs: ribonucleoprotein complexes
RRMs: RNA recognition motifs
SIK2: salt inducible kinase 2
SLC1A5: solute carrier family 1 member 5
SLC2A1: solute carrier family 2 member 1
SLC3A2: solute carrier family 3 member 2
SLC7A11: solute carrier family 7 member 11
SLUG: snail family transcriptional repressor 2
SOX2: SRY-box transcription factor 2
TFs: transcription factors
TMBIM6: transmembrane BAX inhibitor-1-containing motif 6
ULBP2: stress-induced ligands UL16 binding protein 2
USP13: ubiquitin specific peptidase 13
UTR: untranslated region
VEGFA: vascular endothelial growth factor A
WTAP: Wilms tumor 1-associated protein
YTH: YT521-B homology
